# Influence of Drying Conditions on the Durability of Concrete Subjected to the Combined Action of Chemical Attack and Freeze–Thaw Cycles

**DOI:** 10.3390/ma17051131

**Published:** 2024-02-29

**Authors:** Shanshan Song, Hongfa Yu, Haiyan Ma

**Affiliations:** Department of Civil and Airport Engineering, Nanjing University of Aeronautics and Astronautics, Nanjing 210016, China; song332021@163.com

**Keywords:** durability, freeze–thaw cycle, chemical attack, curing condition, fiber, aluminate expansion agent, mineral admixture, salt lake brine

## Abstract

The durability of concrete is critical for the service life of concrete structures, and it is influenced by various factors. This paper investigates the impact of the relative humidity (RH) of the curing environment on the durability of five different concrete types. The aim is to determine a suitable approach for designing concrete that is well-suited for use in the salt lake region of Inner Mongolia. The concrete types comprise ordinary Portland cement (OPC), high-strength expansive concrete (HSEC), high-strength expansive concrete incorporating silica fume, fly ash, and blast furnace slag (HSEC-SFB), steel fiber-reinforced high-strength expansive concrete (SFRHSEC), and high elastic modulus polyethylene fiber-reinforced high-strength expansive concrete (HFRHSEC). All these concrete types underwent a 180-day curing process at three distinct relative humidities (RH = 30%, 50%, and 95%) before being subjected to freeze–thaw cycles in the Inner Mongolia salt lake brine. The curing environment with a 95% RH is referred to as the standard condition. The experimental results reveal that the durability of OPC and HSEC decreases significantly with increasing relative humidity. In comparison with the control sample cured in 95% RH, the maximum freeze–thaw cycles for concrete cured in lower RHs are only 31% to 76% for OPC and 66% to 77% for HSEC. However, the sensitivity of the durability of HSEC-SFB, SFRHSEC, and HFRHSEC to variations in RH in the curing environment diminishes. In comparison with the corresponding reference value, the maximum freeze–thaw cycles for samples cured in dry conditions increase by 14% to 17% for HSEC-SFB and 21% for SFRHSEC. Specifically, the service life of HFRHSEC cured in a low RH is 25% to 46% higher than the reference value. The durability of HSEC-SFB, SFRHSEC, and HFRHSEC has been proven to be appropriate for structures located in the salt lake region of Inner Mongolia.

## 1. Introduction

**Figure 1:**
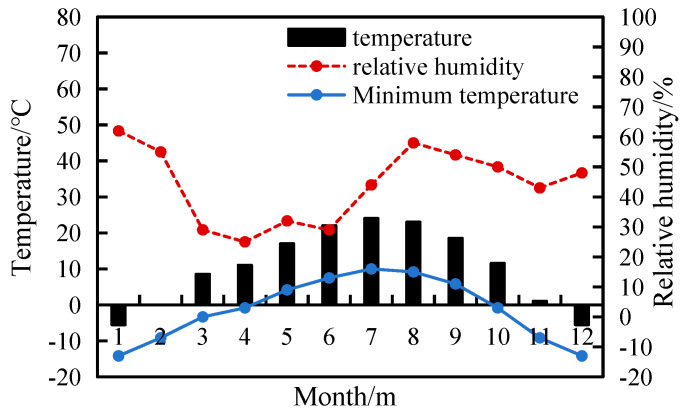
Monthly average temperature and relative humidity in Ordos.

Located in the northwest region of China, numerous salt lakes exhibit diverse compositions and high brine concentrations, leading to significant chemical corrosion and freeze–thaw cycle damage to concrete structures. The Ordos Plateau salt lake area is situated in the southern part of Inner Mongolia. This region encompasses 61 salt lakes, constituting 16.14% of the total statistical salt lakes in the Inner Mongolia [1]. Characterized as an arid to semi-arid desert grassland with low precipitation and relative humidity (RH), this region experiences distinct climatic conditions. Figure 1 illustrates the monthly trends in average temperature and relative humidity in Ordos [2].

Considering the seasonal climatic characteristics of Inner Mongolia, the effects of chemical corrosion and freeze–thaw cycles on concrete are not isolated but occur simultaneously. Many researchers have investigated the durability of concrete under a combined effect of chemical attack and freeze–thaw cycling, achieving significant research outcomes. There is an interaction between freeze–thaw cycling and sulphate erosion [2,3,4,5,6,7,8]. The addition of fly ash (FA), silica fume (SF) [9,10] and recycled brick–concrete aggregates [11] can effectively improve the resistance of concrete to combined freeze–thaw and sulphate erosion. However, a crucial characteristic of the salt lake region of Inner Mongolia is its high environmental temperatures, low RH, high wind speed, extremely dry air, with atmospheric RH generally ranging between 30% and 50%. The dry climate environment not only leads to increased plastic and drying shrinkage of concrete, resulting in noticeable shrinkage cracking, but also raises the permeability of concrete [12,13]. Yu et al. [14] propose that the additions of the composites of SF, FA and blast furnace slag (BFS), super-plasticizer, high-modulus fiber and Aluminate expansion agent (AEA), can reduce the drying shrinkage and improve the moisture sensitivity of concrete shrinkage. To effectively suppress the early plastic shrinkage and cracking of concrete, researchers commonly add fiber-reinforced materials such as polypropylene fibers [15,16,17,18,19,20,21], steel fibers [22], PVA fibers [23], and flax fibers [24]. Lija et al. [25] found that highly absorbent polymeric materials can counteract self-drying shrinkage during concrete hydration. This exacerbates the diffusion and penetration of chloride ions into the concrete [26], thereby significantly impacting its resistance to chemical corrosion and frost resistance [27]. In addition, the latest applications in civil engineering including the bond strength between concrete overlays [28] and the design of sandwich/hybrid cementitious structures [29] are also necessary to consider the effect of the dry environment. Therefore, when studying concrete materials adapted to the salt lake environment in China, it is essential to consider preventing concrete shrinkage cracking and reducing the adverse effects of the dry climate on concrete durability. Ordinary Portland cement (OPC), high-strength expansive concrete (HSEC), and two types of fiber-reinforced high-strength expansive concrete (FRHSEC) were formulated in this paper. The durability of the concrete specimens cured in 30% RH, 50% RH, and 95% RH environments under the damage of chemical corrosion and freeze–thaw cycles in the salt lake brine of Inner Mongolia was measured. Considering the comprehensive effects of various destructive factors, the preparation technology of the high-performance concrete (HPC) for the salt lake regions was discussed.

## 2. Raw Materials and Experimental Methods

### 2.1. Raw Materials

The cement used in this study was P.II 42.5R Portland cement with a Blaine specific surface of 356 m^2^/kg. Its primary mineral composition comprises C_3_S, β-C_2_S, C_3_A, and C_4_AF. Silica fume (SF) was sourced from Qinghai Huadian Ferroalloy Co. (Xining, China), possessing a specific surface area of 20,000 m^2^/kg. Fly ash (FA) grade II, with a screening rate of 0.045 mm and a square hole sieve allowance of 14.4%, manufactured by Qinghai Qiaotou Power Station (Xining, China). The specific surface area of the blast furnace slag (BFS) was 460 m^2^/kg, manufactured by Jiangsu Jiangnan Milling Company (Changzhou, China). The aluminate expansion agent (AEA) primarily consists of binding materials, manufactured by Anhui Chaohu Quick-setting Agent Factory (Chaohu, China), with a chemical composition as detailed in Table 1. The fine aggregate employed was river sand from Nanjing: medium sand, zone II grading, apparent density 2605 kg/m^3^, bulk density 1510 kg/m^3^, compact density 1660 kg/m^3^, void ratio 42%, silt content 1.0%, and fineness modulus 2.75. The coarse aggregate comprised crushed basalt aggregate from Jurong, with a consistent grading of 5–12 mm, an apparent density of 2810 kg/m^3^, an accumulation density of 1600 kg/m^3^, a void ratio 43%, a mud content of 0, a needle sheet particle content of 9.6%, and its crushing index was 2.95%. The high-range water reducer (HRWR) employed was a naphthalene-type superplasticizer powder with a water-reducing percentage exceeding 20%, wherein the Na_2_SO_4_ content was less than 2%, and the Cl^−^ content was below 0.01%, manufactured by Jiangsu Academy of Building Research (Nanjing, China). The steel fiber utilized was a dumbbell-shaped steel fiber manufactured in Jiangxi Engineering fiber Science and Technology Institute (Nanchang, China), having a length of 20 mm and an aspect ratio of 32. The properties of the high-elasticity module polyethylene fiber (HEMPF) manufactured in China are as follows: density of 0.98 kg/m^3^, tensile strength of 2.84 GPa, tensile modulus of elasticity of 73.8 GPa, and elongation at break of 3.8%. Its diameter was 35 µm and length was 20 mm. The water employed in the mixture was tap water.

### 2.2. Mixture Proportions

Five types of concrete were designed, including: (1) OPC with a grade of C30; (2) high-strength expansive concrete (HSEC) with a strength class of C80, incorporating 10% AEA by binder weight; (3) high-strength expansive concrete incorporated with silica fume, fly ash, and blast furnace slag (HSEC-SFB), wherein the additional binder dosage was 10% SF + 20% FA + 20% BFS by binder weight used in HSEC; (4) steel fiber-reinforced HSEC (SFRHSEC) with a strength class of C80 based on the SFS, accounting for 2% of the concrete volume; (5) high-elastic modulus polyethylene fiber-reinforced HSEC (abbreviated as HFRHSEC) with a strength class of C70, accounting for 0.1% of the concrete volume. The specific ratios and performance of the concretes are detailed in Table 2.

### 2.3. Preparation and Cure of Sample

Cement, fine aggregate, coarse aggregate, additives, AEA, HRWR, and fibers were blended for 1 min, followed by a 3 min wet mixing with water. The slump and air content were promptly measured upon discharge. Subsequently, the mixture was poured and vibrated to form prismatic specimens with dimensions of 40 mm × 40 mm × 160 mm. The specimens were subjected to moist curing and the molds were removed after 1 day. The curing process continued in a standard chamber at (20 ± 3) °C and 95% RH for 7 days. Subsequently, the specimens were further cured under three different conditions until reaching a total duration of 180 days for the durability experiments. The three curing conditions maintained a temperature of (20 ± 3) °C, while the RH levels were set at 30%, 50%, and 95%. The dry conditions were controlled using a sealed container with a salt solution [30], resulting in an actual RH of 30.3~31.7% for a saturated CaCl_2_·6H_2_O solution and 49.7~50.8% for a 43% concentration sulfuric acid solution.

### 2.4. Freeze–Thaw Test

Following the “Rapid Freezing Method” stipulated in the standard for test methods of the long-term performance and durability of ordinary concrete (GB/T 50082-2009) [31], the freezing and melting temperatures of the specimens were (−17 ± 2) °C and (8 ± 2) °C. Each freeze–thaw cycle lasted for a duration of 2–4 h, with the melting phase accounting for no less than one-fourth of the entire freeze–thaw period. we employed DTR-1 concrete rapid freeze–thaw testing equipment manufactured by Beijing Yanko New Technology Corporation. The freeze–thaw medium utilized was a simulated brine sourced from the salt lake region of Inner Mongolia. Its chemical composition is outlined in Table 3.

The cycling rule of the freeze–thaw cycles obeys Chinese standard GB/T 50082-2009 [31], the mass of the specimens was determined using an electronic balance with precision of 0.1 g, and the mass change can be calculated based on Equation (1)

(1) $$MC=\frac{m_{0}-m_{t}}{m_{0}}$$

where *m*_0_ is the initial mass of sample (g); *m_t_* is the mass of the sample after the freeze–thaw cycles (g); and *MC* is the mass change of the concrete.

In a study of the effect of freeze–thaw cycles on concrete damage, Ababneh used Equation (1) to define the damage variable of a concrete structure [32]. The dynamic elastic modulus was tested using an NM-4B type non-metallic ultrasonic detector and calculated based on Equation (2) [32]

(2) $$E=\frac{\rho (1+v)(1-2v)}{1-v}V^{2}$$

where *E* is the dynamic elastic modulus; *ρ* is the material density; *v* is Poisson’s ratio of materials; *V* is the ultrasonic velocity of the material. Since Poisson’s ratio and the density of concrete material only result in minor changes, the relative dynamic elastic modulus of the concrete can be calculated by Equation (3) [33].

(3) $$E_{r}=\frac{E_{t}}{E_{0}}=\frac{v_{t}^{2}}{v_{0}^{2}}$$

where *E_r_* is the relative dynamic elastic modulus; *E*_0_ is the initial dynamic elastic modulus of the concrete; *v*_0_ is the ultrasonic speed; *E_t_* and *v_t_* are the dynamic elastic modulus and the ultrasonic speed of concrete that has been subjected to freeze–thaw cycles.

In accordance with GB/T 50082-2009, a specimen is considered to have failed if its relative dynamic modulus falls below 60% or if its mass loss exceeds 5.0%. At this time, the number of freeze–thaw cycles was deemed the maximum number of freeze–thaw cycles that the concrete can withstand, signaling the end of its service life.

### 2.5. Scanning Electron Microscopy (SEM) Observation Experiment

SEM can be employed for the examination of concrete’s microstructure and analysis of its phase composition, overall composition, as well as inclusions. In this experiment, the JSM-5600LV scanning electron microscope (manufactured by Japan Electron Optics Laboratory Co., Ltd., Tokyo, Japan) with a resolution of 3.5 nm under high pressure and 5.0 nm under low pressure at an acceleration voltage of 20 KV was utilized for sample detection and microstructure analysis of the concrete samples. Take the mortar on the concrete surface (0–5 mm) as specimens, and spray gold on the specimen surface before observation.

## 3. Results and Discussions

### 3.1. Durability of OPC

**Figure 2:**
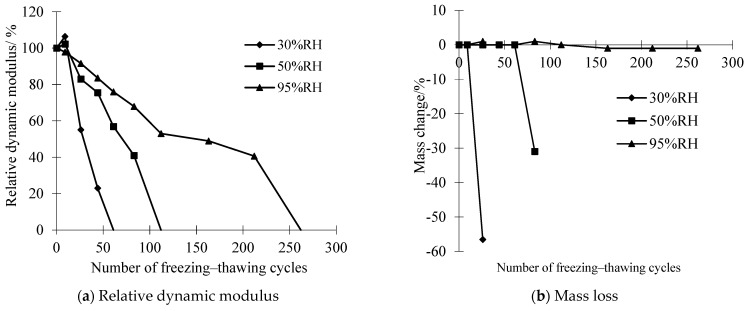
Influence of the RH of the curing environment on the durability of OPC under the simultaneous action of chemical attack and freeze–thaw cycles.

The temporal evolution of the relative dynamic elastic modulus and mass loss in OPC specimens, which were subjected to various curing conditions, is presented in Figure 2. The findings reveal a consistent trend: the coupled impact of chemical attack and freeze–thaw cycles induced a reduction in the relative dynamic modulus (*E_r_*) of all concrete specimens as the number of freeze–thaw cycles increased, concurrent with an elevation in mass loss. This observation aligns with the prior literature [33].

**Figure 3:**
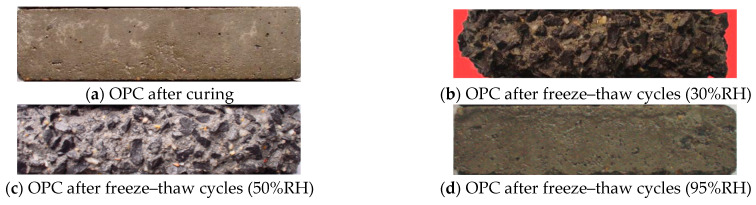
Photos of OPC samples before and after freeze–thaw cycles.

**Figure 4:**
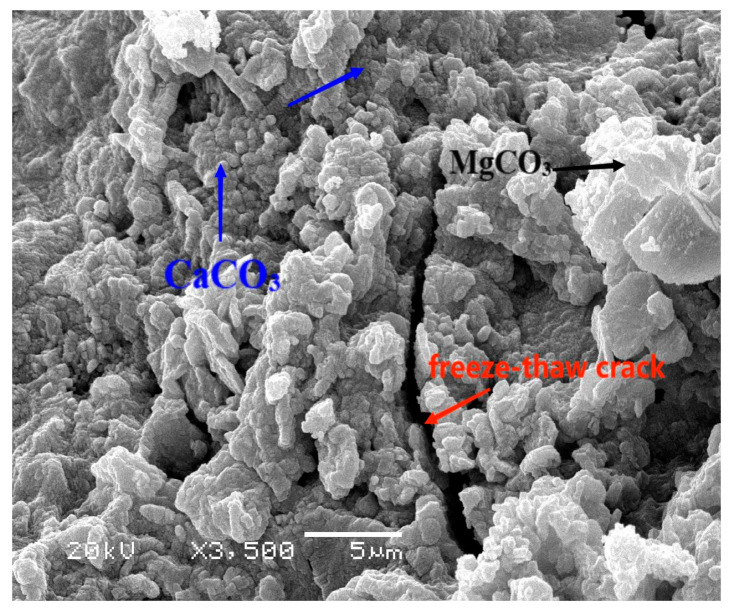
SEM image of OPC samples after 260 freeze–thaw cycles (95% RH).

Comparative analysis of curves corresponding to distinct curing environments highlights an accelerated decline in *E_r_* and mass loss under reduced RH during the curing process. Notably, the distinct divergence between curves denoting different curing environments underscores OPC’s susceptibility to variations in RH. Intriguingly, the introduction of salt to the brine and reducing the freezing point of the solution compared to pure water proves beneficial in mitigating hydraulic pressure during cooling [34]. Moreover, the infiltration of the salt solution into concrete pores leads to supersaturation during the cooling process, resulting in the precipitation of Glauber’s salt crystals (Na_2_SO_4_·10H_2_O) and natron crystals (Na_2_CO_3_·10H_2_O) [4]. The crystallization pressure arising from the growth of these crystals contributes to the progressive deterioration of the concrete [35]. The depicted intricacies in Figure 2 underscore the sensitivity of OPC durability to variations in the RH of the curing environment. Moreover, employing spline fitting on the dataset depicted in Figure 2a, the estimated maximum freezing–thawing cycles for OPC cured at 30% RH, 50% RH, and 95%RH were determined to be 22, 55, and 72. Figure 3 displays sample photographs of OPC before and after undergoing chemical corrosion and freeze–thaw cycles. The red filling mark indicates the freeze–thaw loss of the specimen. Under 95% RH conditions, the degree of surface damage to the OPC is minimal, with essentially no discernible mass loss. According to the results of Tan et al. [36], using XRD and SEM-EDS analysis methods, the chemical corrosion phenomenon exists in OPC, and its corrosion products are mainly calcium carbonate (CaCO_3_), magnesium carbonate (MgCO_3_), and Friedel’s salt (C_3_A·CaC_l2_·10H_2_O). As can be seen from the SEM image in Figure 4, in the inner wall of a capillary hole of OPC there are a lot of low-temperature corrosion products such as CaCO_3_ and MgCO_3_ [33] (the blue arrows indicate CaCO_3_ and the black arrow indicates MgCO_3_), and a micro-crack passes through the pore wall. However, after subjecting OPC to 55 freeze–thaw cycles at 50% RH, observable mortar spalling and coarse aggregate exposure were evident. The end fracture of OPC transpired after 22 freeze–thaw cycles at 30% RH.

These results underscore a notable reduction in the freeze–thaw resistance of OPC when subjected to a simulated salt solution, particularly within the RH range of 30% to 50%. In this context, the maximum number of freezing–thawing cycles for samples decreased by 31% to 76% compared to the reference value, respectively.

### 3.2. Durability of HSEC and HSEC-SFB

**Figure 5:**
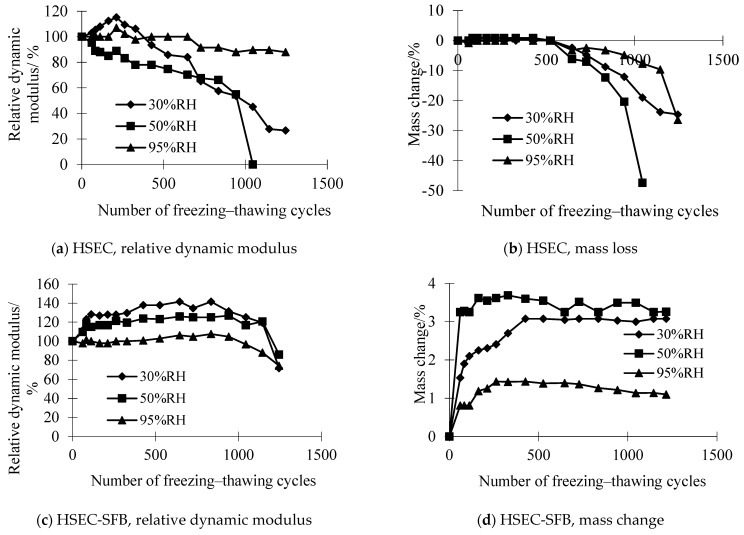
Influence of relative humidity of curing environment on durability of HSEC and HSEC-SFB.

In this section, the impact of three different curing ambient RH (30%, 50%, 90%) on the durability of HSEC and HSEC-SFB under the combined influence of chemical attack and freeze–thaw cycles was investigated. The dynamic elastic modulus and mass variations in HSEC and HSEC-SFB are illustrated in Figure 5. Figure 5a,b reveal a diminishing freeze–thaw resistance of HSEC in a salt solution with decreasing RH of the curing environment. Conversely, Figure 5c,d indicate that the durability of HSEC-SFB experiences an increase with decreasing RH of the curing environment.

**Figure 6:**
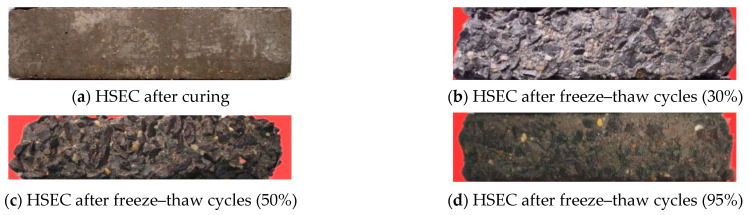
Photos of HSEC samples before and after freeze–thaw cycles.

**Figure 7:**
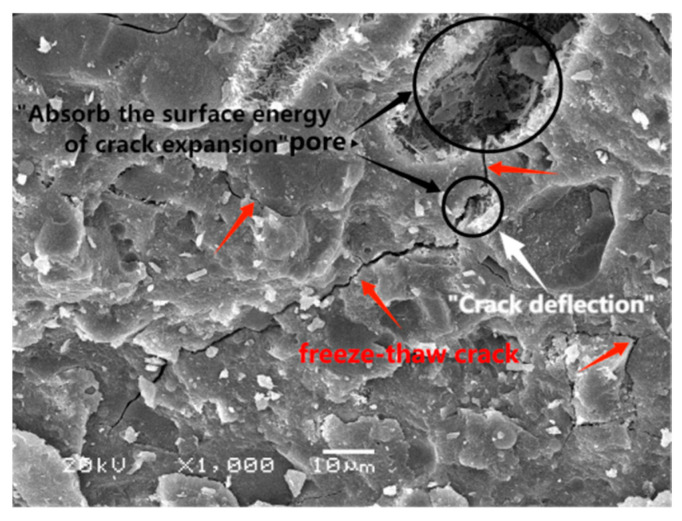
SEM image of HSEC samples after 1025 freeze–thaw cycles (95% RH) [31].

Figure 6 displays images of the HSEC samples after the curing process and the resulting damage following the freeze–thaw cycles. The missing portion of the sample is highlighted in red. In Figure 7, the SEM image of HSEC at 95% RH reveals evident cracks in the sample, and a crack that connects two pores. According to the results of Yu [35] research by XRD and SEM-EDS, low-temperature chemical corrosion products did not appear in HSEC, HSEC-SFB, SFRHSEC, and HFRHSEC, so the microcracks observed by SEM are internal damage caused by 1250 freeze–thaw cycles. The presence of a critical pore can be observed in the SEM image, and the limited number of isolated micron-sized micropores within HSEC function as both channels for absorbing surface energy during frost-induced crack expansion and deflecting microcrack paths, thereby enhancing the durability of high-performance concrete (HPC) in the salt lake region of Inner Mongolia. For HSEC cured under standard conditions at 95% RH, its relative dynamic modulus (*E_r_*) undergoes a gradual decline throughout the freeze–thaw cycles. Simultaneously, mass loss significantly escalates after 850 cycles, reaching the critical value of 5% at 950 cycles. Notably, *E_r_* at failure remains high at 88%, this implies that the failure of HSEC is predominantly attributed to surface scaling, a consequence of micro-crack propagation near the sample surface [4]. Contrastingly, HSEC samples cured at 50% RH exhibit a noticeably lower *E_r_* compared to the reference, with a faster degradation rate. Additionally, mass loss for HSEC cured at 50% RH significantly increases after 550 freeze–thaw cycles. At 650 cycles, its mass loss and *E_r_* stand at 6.1% and 70%, respectively. The HSEC sample cured at 30% RH demonstrates an increase in *E_r_* during the early freeze–thaw cycles, a phenomenon that can be explained as follows.

Under 50% RH conditions, owing to the low water-binder ratio, the AEA undergoes incomplete hydration, and a portion of the unhydrated AEA continues to react during the freeze–thaw cycles. The monosulfide hydrated calcium sulfoaluminate (AFm) produced through hydration will subsequently transform into ettringite (AFt), and the expansion pressure generated during ettringite formation leads to concrete cracking [37]. Consequently, there is a significant drop in the dynamic elastic modulus, accompanied by mass loss. The anhydrous cement content in HSEC cured at 30% RH is relatively high. During the initial freeze–thaw cycles, anhydrous cement reacts with the penetrated solution’s water, producing hydration products. The main hydration products fill concrete pores more effectively than anhydrous cement [34], leading to an improved microstructure. When the enhanced microstructure outweighs the freeze–thaw damage, the sample’s relative dynamic modulus increases. The sample’s Er reaches a peak of 115% after 115 freeze–thaw cycles and then decreases rapidly, with a deterioration rate much higher than that of the reference sample cured at 95% RH. Spline fitting analysis reveals that the maximum freeze–thaw cycles for HSEC samples cured at 30% RH, 50% RH, and 95% RH are 734, 624, and 949. These data indicate that the service life of HSEC cured at lower RH values is 66% to 77% of the reference value.

Therefore, HSEC’s freeze–thaw resistance in a salt solution mimicking Inner Mongolia’s salt lake brine decreases with curing environment RH. In essence, concrete durability is responsive to RH changes during curing. When compared to Figure 2, these results demonstrate superior performance to OPC under similar conditions.

For HSEC-SFB cured at 95% RH, the penetration of brine during freeze–thaw cycles may damage the sample. However, the interaction between the water in the brine and the reactive mineral admixtures can lead to reactions with CH released from anhydrous cement hydration. As hydration dominates the concrete microstructure evolution, *E_r_* gradually increases before reaching 950 freeze–thaw cycles, despite the brine penetration. The cycles also increase the mass of the concrete specimens. After a certain number of cycles, freeze–thaw damage and chemical attack become dominant, causing a slow reduction in *E_r_* and a slight mass loss, yet mass still remains greater than the initial value. For instance, at 1250 freeze–thaw cycles, the *E_r_* of the HSEC-SFB sample is 74%, with a 1.1% mass increase. This phenomenon highlights the distinct durability behavior of HSEC-SFS compared to HSEC under the same curing conditions.

Furthermore, Figure 5c,d indicate that the durability of HSEC-SFB improves with decreasing RH in the curing environment. This is attributed to the coarsening of the pore structure in the dry curing environment, leading to increased salt solution absorption and sample mass. However, saturation occurs after 300–500 freeze–thaw cycles. Thus, for HSEC-SFB cured in a dry environment, the concrete microstructure improves due to the combined effects of chemical attack and freeze–thaw cycles, facilitated by salt crystallization in pores and continuous mineral admixture hydration [24]. Spline fitting on the curves in Figure 5c predicts the maximum freeze–thaw cycles for HSEC-SFB cured at 30% RH, 50% RH, and 95% RH to be 1357, 1386, and 1189. Notably, the former two are 14% to 17% higher than the latter.

Under the combined effects of chemical attack and freeze–thaw cycles, the durability of HSEC-SFB remains unaffected by the RH of the curing environment. Therefore, HSEC-SFB proves suitable for use in the salt lake environment of Inner Mongolia.

### 3.3. Durability of SFRHSEC and HFRHSEC

**Figure 8:**
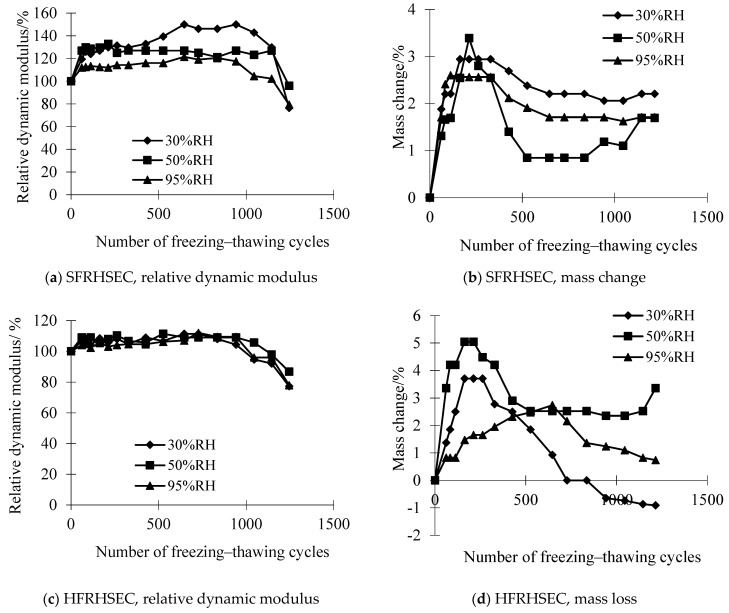
Influence of relative humidity of curing environment on durability of SFRHSEC and HFRHSEC.

**Figure 9:**
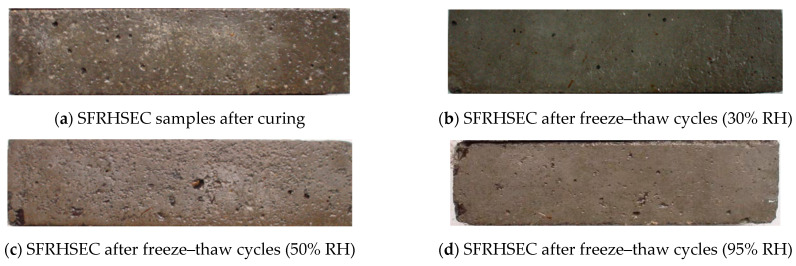
Photos of SFRHSEC samples before and after freeze–thaw cycles.

**Figure 10:**
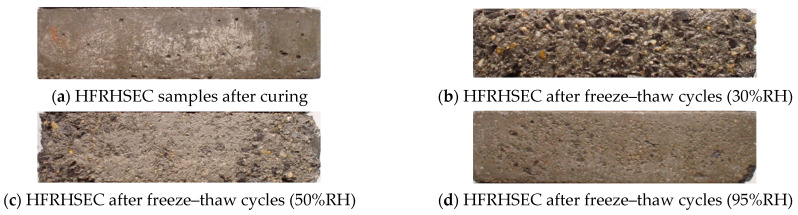
Photos of HFRHSEC specimens before and after freeze–thaw cycles.

**Figure 11:**
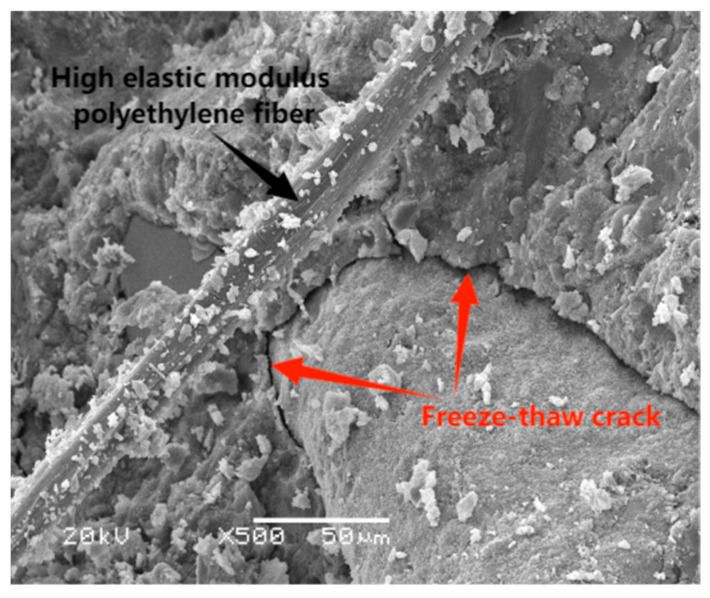
SEM image of HFRHSEC specimen after freeze–thaw cycles (95% RH).

This section examines the durability of two types of fiber-reinforced high-strength, environmentally friendly concrete (HSEC) when subjected to simultaneous chemical attack and freeze–thaw cycles. The first type is steel fiber-reinforced HSEC (SFRHSEC) and the second type is high-elastic modulus polyethylene fiber-reinforced HSEC (HFRHSEC). Figure 8 depicts the dynamic elastic modulus and mass loss evolution of the concretes over time at different relative humidities of the curing environment (30% RH, 50% RH, and 95% RH). Figure 9 and Figure 10 depict photos of SFRHSEC and HFRHSEC specimens before and after the freeze–thaw cycles, respectively. Following the freeze–thaw process, noticeable surface roughness and mortar spalling are observed in the samples. Additionally, Figure 11 presents SEM images of HFRHSEC after experiencing freeze–thaw failure under 95% RH. It is noteworthy that the two cracks are interconnected to form a U-shape, and they do not extend in the original direction. This observation suggests that the fibers can effectively impede crack propagation.

The results indicate that the trends of *E_r_* for SFRHSEC and HFRHSEC are similar to those of HSEC-SFB. Initially, the mass of all samples increases during the freeze–thaw cycles and then gradually decreases. Estimation based on the curves in Figure 8a reveals maximum freezing–thawing cycle values for SFRHSEC cured at 30% RH, 50% RH, and 95% RH as 1364, 1369, and 1123. This implies that the service life of SFRHSEC increases by 15% to 21% when cured at a low RH compared to the reference sample. Using a similar approach, the maximum freezing–thawing cycle values for HFRHSEC are 1519 at 30% RH, 1295 at 50% RH, and 1035 at 95% RH. For HFRHSEC cured at 30% RH, its maximum freezing–thawing cycle value is as much as 12% higher than that of HSEC-SFB under the same conditions. The service life of HFRHSEC in a low-RH environment increases by 25% to 46% compared to the reference case.

In summary, the durability of SFRHSEC and HFRHSEC will not become compromised under the combined effects of chemical attack and freeze–thaw cycles in a dry environment. On the contrary, their service life generally increases. The results demonstrate that appropriate selection of raw materials and a proper mixture design can mitigate the negative impact of low RH on the structural service life of concrete.

### 3.4. Relationship between RH and Service Life of Concretes

**Figure 12:**
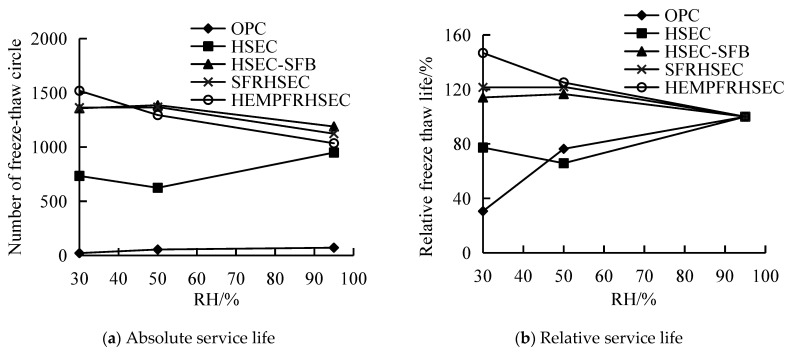
Comparison of service life of different types of concrete in various relative humidity of curing environment.

To clarify the influence of RH on the service life of concrete and to identify differences in service life between different types of concrete, we collected and plotted the predicting values from the previous sections in Figure 12. Figure 12a shows the relationship between the predicting value of the maximum number of freeze–thaw cycles (absolute service life) and the RH of the curing environment. Figure 12b displays the curve of the relative service life of concrete in relation to the RH of the curing environment. The relative service life is defined as the maximum freeze–thaw cycles of concrete cured in specified RH divided by that of concrete cured in a 95% RH.

Evidently, it can be seen in Figure 12 that the low RH of the curing environment shortens the service lives of OPC and HSEC. On the contrary, the service lives of HSEC-SFB and SFRHSEC increase with the decreasing RH of the curing environment. And under the condition of a 30% RH curing environment, the RH-sensitivity of the durability of HSEC is smaller than that of OPC. Under the lower curing RH (30% RH and 50% RH), the comparison of the relative service lives of HSEC-SFB, SFRHSEC, and HFRHSEC demonstrates that incorporation of fiber may further improve the durability of concrete in cured in a dry environment. Thus, HSEC-SFB, SFRHSEC, and HFRHSEC have excellent durability under the simultaneous actions of freeze–thaw cycles and the simulated brine of the salt lake region of Inner Mongolia. They are especially suitable for application in the dry environments of salt lake regions. The above experiment results on the durability of various types of concrete provide ample information on the preparation techniques for HPC in salt lake regions.

## 4. Conclusions

(1) The durability of OPC and HSEC is adversely affected by the dry curing environment when subjected to freeze–thaw cycles in a simulated brine solution representative of a salt lake in Inner Mongolia. The rate of deterioration of both OPC and HSEC increases as the RH of the curing environment decreases. Some specimens even experienced structural collapse during the freeze–thaw cycles. Based on reference values, the freeze–thaw service life of OPC and HSEC cured in the range of 30% RH to 50% RH corresponds to only 31% to 76% and 66% to 77%. Furthermore, the freeze–thaw service life of HSEC in the Inner Mongolia salt lake brine demonstrates less sensitivity to variations in the RH of the curing environment when compared to OPC.

(2) In dry conditions, the freeze–thaw resistance of HSEC-SFB, SFRHSEC, and HFRHSEC in Inner Mongolia salt lake brine is insensitive to variation in RH in the curing environment. The concrete’s microstructure is enhanced through a combination of salt crystallization within pores and mineral admixture hydration during the freeze–thaw cycles. As a result, any damage caused by these cycles is outweighed by the improvement in microstructure. This leads to an increase of 14% to 17% in the maximum number of freezing–thawing cycles for HSEC-SFB cured in a dry environment compared to the reference sample cured under standard conditions. Additionally, when SFRHSEC and HFRHSEC are cured in a dry environment, their freeze–thaw service life increases by 21% and 25% to 46%, respectively, compared to their corresponding reference values.

(3) Under the coupling actions of freeze–thaw cycles and chemical attack by Inner Mongolia salt lake brine, HSEC-SFB, SFRHSEC, and HFRHSEC exhibit excellent durability. Consequently, these three types of concrete prove to be particularly well-suited for applications in the dry environments of salt lake regions where both chemical attack and frost damage are prevalent. Thus, in dry salt lake regions, the recommended fundamental approach to HPC preparation involves the judicious combination of shrinkage-compensation techniques (utilizing expansion agents), water-reducing techniques (employing superplasticizers), the incorporation of various types of mineral admixtures (such as fly ash, blast furnace slag, silica fume), and the integration of fiber reinforcing techniques.

## Figures and Tables

**Table 1:** Chemical composition of raw materials (wt%) [14].

Sample	SiO_2_	Al_2_O_3_	CaO	MgO	SO_3_	Fe_2_O_3_	MnO	TiO_2_	Na_2_O	K_2_O	I.O.L.
Cement	20.60	5.03	65.06	0.55	2.24	4.38	--	--	--	--	1.30
SF	85.16	0.06	0.56	2.10	--	0.75	--	--	--	--	2.68
FA	58.30	20.97	5.16	1.46	0.81	7.04	--	--	--	--	2.64
BFS	34.20	14.2	41.70	6.70	1.00	0.43	0.30	1.07	1.27	0.56	1.70
AEA	19.82	16.62	28.60	1.58	26.86	2.66	--	--	0.32	0.30	3.02

**Table 2:** Mixture proportions and performance of concretes.

	OPC	HSEC	HSEC-SFB	SFRHSEC	HFRHSEC
Cement (kg·m^−3^)	325	540 (90%)	270 (45%)	270 (45%)	270 (45%)
Silica fume (kg·m^−3^)	0	0	54 (9%)	54 (9%)	54 (9%)
Fly ash (kg·m^−3^)	0	0	108 (18%)	108 (18%)	108 (18%)
blast furnace Slag (kg·m^−3^)	0	0	108 (18%)	108 (18%)	108 (18%)
AEA (kg·m^−3^)	0	60 (10%)	60 (10%)	60 (10%)	60 (10%)
Fine aggregate (kg·m^−3^)	647	610	610	785	785
Coarse aggregate (kg·m^−3^)	1150	1134	1134	957	957
Water (kg·m^−3^)	195	150	172	172	172
HRWR (kg·m^−3^)	0	3.9	3.9	5.0	6.5
Steel fiber (kg·m^−3^)	0	0	0	156	0
HEMPF (kg·m^−3^)	0	0	0	0	1
Slump (mm)	45	45	45	35	45
Air content (%)	1.4	1.8	2.0	2.1	3.0
W/B	0.6	0.25	0.28	0.28	0.28
180 d flexural strength (MPa)	8.06	14.85	13.06	24.51	10.57

**Table 3:** Chemical compositions of the brine in Inner Mongolia salt lakes.

Na^+^ (mg·L^−1^)	Mg^2+^ (mg·L^−1^)	K^+^ (mg·L^−1^)	Ca^2+^ (mg·L^−1^)	Cl^−^ (mg·L^−1^)	SO_4_^2−^ (mg·L^−1^)	CO_3_^2−^ (mg·L^−1^)	HCO_3_^−^ (mg·L^−1^)	pH
97,167.92	3671.00	2638.42	129.29	107,790.00	36,445.42	25,382.08	4595.42	10

## Data Availability

Data are contained within the article.
